# Organic Semiconductor Single Crystal Arrays: Preparation and Applications

**DOI:** 10.1002/advs.202300483

**Published:** 2023-03-26

**Authors:** Xiaotong Zhao, Hantang Zhang, Jing Zhang, Jie Liu, Ming Lei, Lang Jiang

**Affiliations:** ^1^ State Key Laboratory of Information Photonics and Optical Communications & School of Integrated Circuits Beijing University of Posts and Telecommunications Beijing 100876 China; ^2^ Beijing National Laboratory for Molecular Sciences Key Laboratory of Organic Solids Institute of Chemistry Chinese Academy of Sciences Beijing 100190 China; ^3^ College of Chemistry and Material Science Shandong Agricultural University Taian 271018 China; ^4^ School of Chemistry and Materials Science of Shanxi Normal University & Key Laboratory of Magnetic Molecules and Magnetic Information Materials of Ministry of Education Taiyuan 031000 China

**Keywords:** Integrated circuits, organic field‐effect transistors, organic semiconductor single crystal arrays

## Abstract

The study of organic semiconductor single crystal (OSSC) arrays has recently attracted considerable interest given their potential applications in flexible displays, smart wearable devices, biochemical sensors, etc. Patterning of OSSCs is the prerequisite for the realization of organic integrated circuits. Patterned OSSCs can not only decrease the crosstalk between adjacent organic field‐effect transistors (OFETs), but also can be conveniently integrated with other device elements which facilitate circuits application. Tremendous efforts have been devoted in the controllable preparation of OSSC arrays, and great progress has been achieved. In this review, the general strategies for patterning OSSCs are summarized, along with the discussion of the advantages and limitations of different patterning methods. Given the identical thickness of monolayer molecular crystals (MMCs) which is beneficial to achieve super uniformity of OSSC arrays and devices, patterning of MMCs is also emphasized. Then, OFET performance is summarized with comparison of the mobility and coefficient of variation based on the OSSC arrays prepared by different methods. Furthermore, advances of OSSC array‐based circuits and flexible devices of different functions are highlighted. Finally, the challenges that need to be tackled in the future are presented.

## Graphical abstract

In this review, the general strategies for patterning organic semiconductor single crystals (OSSCs) are summarized, especially, patterning of monolayer molecular crystals (MMCs) is emphasized. Then, organic field‐effect transistor (OFET) performance is generalized based on the OSSC arrays. Furthermore, advances of OSSC array‐based applications are highlighted. Finally, the challenges that need to be tackled in the future are presented.

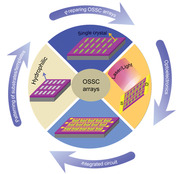

## 1 Introduction

Compared to inorganic semiconductors, organic semiconductors have many prevalent non‐negligible advantages, such as biocompatibility, lightweight and low‐temperature processing, molecular structure tailored properties and high compatibility with various types of substrates, etc. which endow them indispensable status in flexible and transparent plastic electronics. The development of modern electronics has promoted their related applications in optoelectronics,^[^ ^1^, ^2^, ^3^ ^]^ energy storage,^[^ ^4^, ^5^ ^]^ integrated circuits,^[^ ^6^, ^7^ ^]^ wearable sensors^[^ ^8^, ^9^ ^]^ and so on. As one of the basic components in organic circuits, organic field‐effect transistor (OFET) has attracted broad research attention since the 1980s.^[^ ^10^ ^]^ In the past few decades, the mobility of OFETs has increased from 10^−6^–10^−5^ cm^2^ V^−1^ s^−1^ to 10–40 cm^2^ V^−1^ s^−1^ by designing new materials and optimizing the device fabrication process.^[^ ^11^, ^12^, ^13^, ^14^, ^15^, ^16^, ^17^, ^18^, ^19^, ^20^ ^]^ It has been proved that the semiconductor morphology and molecular packing have crucial impacts on their OFET properties,^[^ ^21^, ^22^, ^23^ ^]^ and organic semiconductor single crystals (OSSCs) are usually found with higher mobility than thin films because of the absence of grain boundaries and reduced trap density.^[^ ^24^ ^]^ Moreover, OSSC is regarded as a powerful tool for revealing the intrinsic properties of organic semiconductors, understanding structure‐property relationships and exploring device physics.^[^ ^25^ ^]^

**Figure 1:**
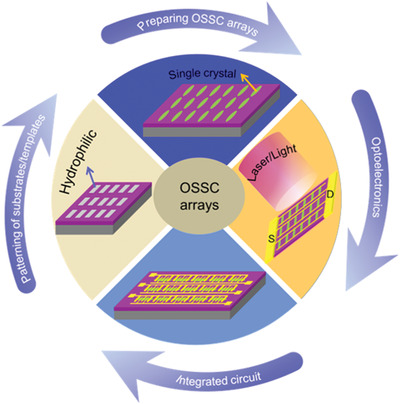
Schematic showing the main contents.

Despite the numerous advantages of OSSC electronics,^[^ ^26^, ^27^, ^28^ ^]^ the application of OSSC in organic integrated circuits and flexible electronic devices relies on two critical issues: controllable OSSC growth, including uniform orientation and identical thickness; and patterning of OSSCs at desired location. Given the remarkable anisotropy of the charge transport properties of OSSCs, the mobilities may vary several times along different crystal axis.^[^ ^29^, ^30^ ^]^ Thus, the preparation of OSSCs with uniform orientation would help to narrow the mobility distribution. Despite crystal orientation, OSSCs with identical thickness would guarantee uniform series resistance, which would further promise small variable coefficient of the key parameters in OFET arrays. Patterning of OSSCs can reduce the gate‐source leakage current and cross‐talk caused by drain–source fringe current. The patterning of OSSCs would also makes it easier to integrate OFETs with other device elements. The growth of OSSCs undergoes two processes: nucleation and growth. Unlike inorganic crystals, in OSSCs, organic molecules are bounded by weak van der Waals force and have low tolerance to high‐energy rays, high temperature and organic solvents, making them unsuitable for traditional lithography patterning techniques.^[^ ^31^, ^32^ ^]^ In particular, the fabrication of OSSC arrays usually requires strong intervention in the nucleation and/or crystal growth process. Up to now, tremendous efforts have been devoted in the controllable preparation of OSSC arrays, and great progress has been achieved either in morphology control or in OFET performance.^[^ ^18^, ^19^, ^20^, ^33^, ^34^, ^35^ ^]^ Moreover, monolayer molecular crystals (MMCs), a recently emerged novel form of OSSCs, offers an alternative solution to the performance homogeneity issue of OSSC device arrays due to its uniform active layer thickness.^[^ ^36^ ^]^ However, there are few review articles on single crystal patterning. Therefore, it is urgent to provide a comprehensive summary of the patterning techniques developed for the preparation of OSSC arrays and applications of these arrays in OFET‐based circuits (**Figure** **1**).

In this review, the recent progress of preparing OSSC patterns are summarized. Firstly, a brief overview of prepatterning of substrates/templates is given. This is followed by an emphasis on the state‐of‐the‐art strategies for the construction of aligned/patterned OSSC arrays in details, including the patterning of MMCs. Then, the mobility and coefficient of variation (CV) of OFET performance are extracted from different materials and processing techniques. Finally, applications of OSSC arrays are stressed. The challenges of OSSC arrays are provided in the end. We hope that this review would give an overview of the state‐of‐art status and guide future work in this area.

## 2 Methods for Preparing OSSC Arrays

The spontaneous growth of OSSCs leads to disorder in orientation and thickness. Therefore, additional forces need to be artificially introduced to interfere with the growth process of OSSCs to obtain OSSC arrays. The introduction of additional forces always starts with the substrates/templates pretreatment, i.e., hydrophobic/hydrophilic patterns or/and surface microstructure construction, followed by physical vapor transport (PVT) or solution processes to complete the OSSC array growth. In this section, the common prepatterning methods of substrates/templates are firstly introduced, then the PVT/solution techniques adopted for OSSC‐array growth are summarized.

### 2.1 Prepatterning of Substrates/Templates

**Figure 2:**
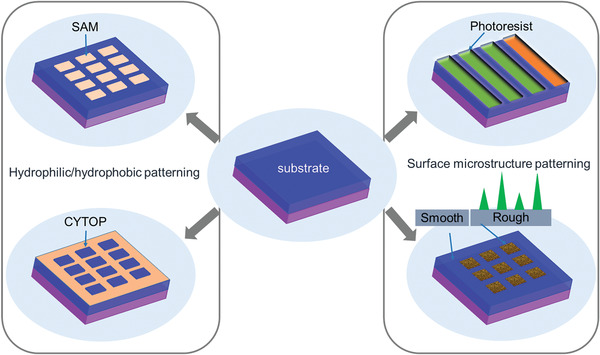
Schematic illustration of the typical hydrophilic/hydrophobic and surface microstructure patterning of substrates/templates.

Patterning of OSSCs could be realized by selected crystal growth either by PVT method or solution processing techniques on prepattern substrates or under the assistance of prepatterned templates. The patterns of substrates/templates could be classified into hydrophobic/hydrophilic patterns and surface microstructure patterns as illustrated in **Figure** **2**. In this section, we will summarize the preparation of such patterns in brief.

#### 2.1.1 Hydrophilic/Hydrophobic Patterning of Substrates/Templates

To achieve hydrophobic/hydrophilic patterns on substrates/templates, firstly, hydrophobic modification should be carried out. The most widely used hydrophobic material is self‐assembled monolayers (SAMs) of octadecyltrichlorosilane (OTS).

**Figure 3:**
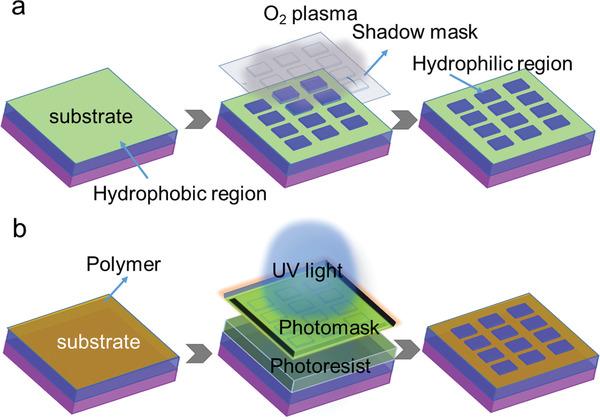
Schematic illustration of a) typical hydrophobic/hydrophilic patterns or surface microstructure patterns; b) the photolithography process for preparing surface microstructure patterns.

Besides, composite SAM layer of tetramethoxysilane (TMS) and decyltrimethoxysilane (DTMS) can also be used to pattern hydrophobic/hydrophilic region.^[^ ^37^ ^]^ After the deposition of OTS SAMs on hydrophilic substrate, O_2_ plasma treatment is then conducted under a shadow mask with desired patterns. Then hydrophilic patterns embedded in hydrophobic OTS substrates are obtained (**Figure** **3**a). And further deposition of organic semiconductor solution would result in selected growth of OSSCs on hydrophilic regions. Elaborately designed patterns with reasonable sizes and shapes can even modulate the nucleation concentration and adjust the crystal orientation.^[^ ^38^ ^]^ Besides, by depositing gold strips on perfluoro(1‐butenylvinylether) polymer (CYTOP) dielectrics could also realize the hydrophobic/hydrophilic patterns. By adopting proper processing techniques, such as blade coating,^[^ ^39^ ^]^ such patterns could guide solution‐controlled spreading on hydrophobic CYTOP, and finally realize the whole coverage of OSSCs on and between electrode pairs.

#### 2.1.2 Surface Microstructure Patterning of Substrates/Templates

It is known that, substrate with different surface energies would result in different growth mode, nucleation and crystal growth would preferentially happen at surface with larger surface energy. Thus, intentional introduction of structures with reasonable roughness would realize selective crystal nucleation and growth. For example, Bao et al. developed a polydimethylsiloxane (PDMS) stamp with the desired pattern‐relief structure, after impressing the stamp onto a clean SiO_2_/Si substrate by microcontact printing thick OTS films (mean thickness, ≈13 nm), rough OTS patterns with defined size and location are then formed.^[^ ^40^ ^]^ This method is proven quite effective in realizing OSSC pattern through PVT method and will be discussed in Section 2.2.

In the solution process, the semiconductor molecules are carried by solvents. Thus, surface microstructures on substrates or templates can define the wetting behavior and therefore realize controlled OSSC growth on desired location. Microstructures such as microchannels on substrates and micropillars embedded in templates could be applied for the growth of OSSC arrays. A typical example of constructing microchannels on substrate is shown in Figure 3b, the combination of the microstructured substrates and proper solution processing can realize highly uniform, aligned OSSC arrays.^[^ ^41^, ^42^, ^43^ ^]^ Besides, groove structures on substrates, by pressing a template with patterned structures on top of the substrates, periodical pin effect is then formed, which would result in directional contraction of the solution and realize the growth of OSSC arrays.^[^ ^44^ ^]^ The prepatterns of substrates or utilization of templates could assist the selective nucleation and growth of OSSCs on desired locations, however, OSSC patterns with controlled orientations could not be realized unless proper processing techniques are developed.

Based on the physicochemical properties of organic semiconductor materials, the most widely used methods for OSSC growth are PVT and solution processes. In contrast, the PVT method usually offers the optimal outcome for OSSCs with high quality, high chemical purity and higher optoelectronic properties. However, solution processing is easier to manufacture on a large scale at low cost and therefore holds great promise for practical applications. The combination of various prepatterned substrates/templates and diverse single crystal preparation methods leads to abundant techniques for the preparation of OSSC arrays. In the following sections, the techniques developed in the recent years are summarized with an emphasize on the assessment of the uniformity of crystal orientation and thickness of the obtained OSSC arrays.

### 2.2 PVT for Patterning OSSC Arrays

**Figure 4:**
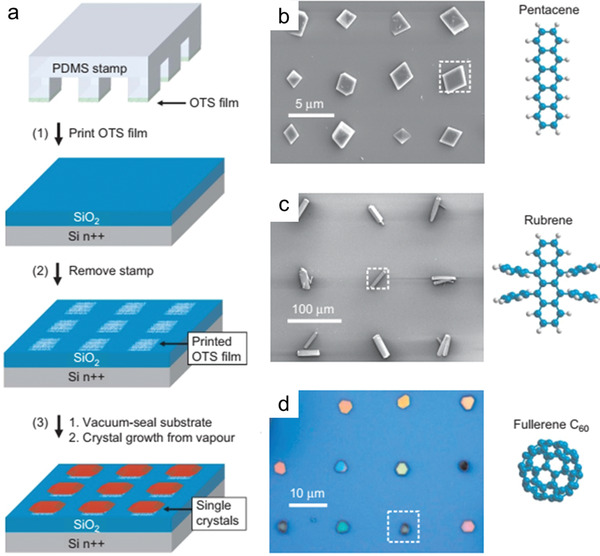
a) Schematic outline of the procedure used to grow OSSCs on substrates that have been patterned by microcontact printing. SEM b,c) and optical micrograph d) of patterned single‐crystal arrays of pentacene, rubrene and fullerene C_60_. The dotted square in each image indicates the size and location of one of the OTS‐stamped domains. Reproduced with permission.^[^ ^40^ ^]^ Copyright 2006, Springer Nature.

Compared with the solution processing techniques, the study of patterning OSSCs in PVT process is limited. As one typical successful example, Bao et al. adopted substrates with prepatterned rough OTS domains, the rough OTS pattern behave as effective primary nucleation sites (**Figure** **4**a).^[^ ^40^ ^]^ After the film is deposited, the substrate was placed at the crystal growth zone for the PVT process, then crystals with controlled density and defined location were obtained. The universality of this method is proved by a variety of materials (Figure 4b–d), including high mobility p‐type rubrene, pentacene and tetrobenzene, and n‐type materials such as C60, copper fluoride phthalocyanine (F_16_CuPc) and tetrachyanoquinone dimethane (TCNQ). Despite the well‐controlled density and location, this method cannot guarantee identical crystal orientation and thickness. Besides substrate modification, the different interactions between semiconductor/electrode and semiconductor/dielectric could also realize the selective nucleation and growth of OSSC on electrodes.^[^ ^45^ ^]^ For example, Chi et al. demonstrated the preparation of demtriethylsilylethynyl anthradithiophene (TES‐ADT) OSSC arrays on substrates with bottom electrode pairs. In this setup, firstly, amorphous/polycrystalline films covering electrode pairs were obtained after vapor deposition at substrate temperature of 155 °C, due to the stronger interaction of molecules with Au in comparison to that with SiO_2_. Subsequent solvent vapor annealing (SVA) process could assist the rearrangement of the molecules and OSSCs self‐aligned on the Au microelectrode pairs are obtained. Though PVT and vacuum evaporation are readily applicable technologies for most of the small‐molecule organic semiconductors, the thickness of the OSSCs prepared by these methods is not uniform and the crystal orientations are arbitrary.

### 2.3 Solution Processing Techniques for Patterning OSSC Arrays

The good solubility of organic semiconductors in common organic solvents renders a variety of solution‐based manufacturing techniques. Compared with PVT and vacuum evaporation, solution processing techniques are usually carried out in relatively mild conductions, such as atmospheric environment at low temperature (below 90 °C). Besides, mass‐production could be realized in solution‐process, and large‐scale films or OSSCs have been successfully demonstrated. More importantly, in the OSSCs patterning process, more parameters could be introduced and modified to regulate the OSSC nucleation and growth. Because of the distinctive properties of solution processing techniques, novel strategies have been developed for OSSC patterns with improved orientation identity. These strategies include, but not limited to, controlling the fluid flow, tuning the solvent composition by adding of antisolvent or soluble additives, controlling evaporation rate using asymmetric patterns, contact line engineering, alignment control via meniscus guide, etc.

#### 2.3.1 Inkjet Printing

**Figure 5:**
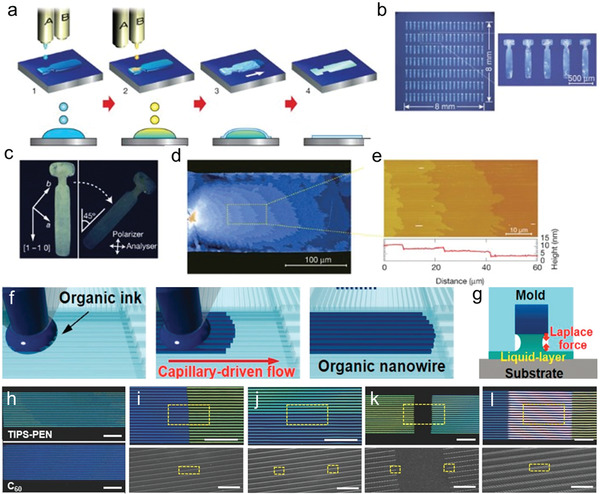
a) Schematic of inkjet printing of organic single‐crystal thin films. Antisolvent ink A) is first inkjet‐printed (step 1), and then solution ink B) is overprinted sequentially to form intermixed droplets confined to a predefined area (step 2). Semiconducting thin films grow at liquid–air interfaces of the droplet (step 3), before the solvent fully evaporates (step 4). b) Optical micrographs (OM) of a 20×7 array of inkjet‐printed C8‐BTBT single‐crystal thin films. c) POM of the film. d) Expanded micrograph of the film. e) AFM image and the height profile (below). Reproduced with permission.^[^ ^18^ ^]^ Copyright 2011, Springer Nature. f) Inkjet‐assisted nanotransfer printing (inkjet‐NTP). Schematic illustration of an ink droplet filling the recessed nanochannels of a selected area of the mold through capillary‐driven flow. g) Schematic illustration of a liquid bridge formed by a polar liquid layer between the nanowires and a substrate. h–l) Nanopatterns composed of 6,13‐bis(triisopropylsilylethynyl) pentacene (TIPS‐PEN), C60; heterojunction nanowires. The POM images demonstrate the structures of the nanopatterns (scale bars: 30 µm). i–l) The SEM images show the alignment and morphology of each nanowire (scale bars: 15 µm (top) and 100 nm (bottom)). Reproduced with permission.^[^ ^46^ ^]^ Copyright 2016, Wiley‐VCH.

Inkjet printing is a prospering technology for fabricating large‐scale organic electronic devices because of its material‐saving, low‐cost, and on‐demand features. Nevertheless, during the solidification of a droplet, the unavoidable “coffee ring” effect usually results in discontinuous film shapes, and this phenomenon could be effectively relieved by antisolvent‐assisted crystallization. In 2011, Hasegawa et al. have used a dual‐head printer to generate small molecular single crystalline films under the principle of antisolvent crystallization on a prepatterned hydrophilic/hydrophobic substrate.^[^ ^18^ ^]^ As shown in **Figure** **5**a, an antisolvent droplet is first deposited prior to the printing of an organic semiconductor ink droplet. Then, crystal nucleation and growth take place during the solvent evaporation above the antisolvent liquid surface. Finally, crystals with different layer thicknesses are obtained on the substrate after the complete evaporation of the antisolvent. It should be noted that, the elaborately designed hydrophilic area containing a protuberance was proved effective in regulating the crystal orientation. By carefully optimizing the deposition parameters, such as the substrate temperature, solvent volume, solvent–antisolvent ratio, and shape of the droplets, more than half of the crystals have the same orientation and show a single domain over the entire pattern. From the microscope images of the films shown in Figure 5b–d, we can see crystalline films with uneven thickness and intervals of several micrometers to several tens of micrometers are obtained. And the step‐height is estimated to be about 2.6–2.8 nm (Figure 5e) as evidenced by atomic‐force microscopy (AFM), which associated well with the height of the molecular step of 2,7‐dioctyl[1]‐benzothieno[3,2‐b][1]benzothiophene (C8‐BTBT). The combination of a solvent‐antisolvent with a predefined patterns of designed hydrophilic area proves to be effective in producing OSSC arrays. However, improvement is needed for further optimization of the orientation and thickness.

Besides, Park et al. adopted the inkjet printing process under the assistance of a mold with nanochannels, as shown in Figure 5f. After the printing of semiconductor solutions, the capillary force provided by the mold would assist the formation of single‐crystalline nanowires after drying process at elevated temperature. The method also facilitates the heterogeneous monolithic integration of various patterns with diverse organic semiconductors on a single substrate. Furthermore, by precisely controlling the number and volume of droplets, the length and number of nanowires can be adjusted, respectively. After the formation of single crystal nanowires in the mold, all nanowires can be simply transferred by applying a thin liquid layer between the mold and the substrate to form a capillary bridge that causes the nanowires to leave the mold and eventually transfer to the substrate (Figure 5g). This approach allows the integration of various organic nanopatterns by sequentially printing different materials.^[^ ^46^ ^]^ The orientation and morphology of OSSC arrays obtained by this method is improved as evidenced by the polarization optical microscope (POM) and/or scanning electron microscopy (SEM) images of the various nanopatterns printed on the wafer (Figure 5h–l).

The “coffee ring” effect could also induce in incomplete coverage of OSSCs on patterns, which can be optimized by SVA process.^[^ ^47^ ^]^ However, the liquid crystalline nature of semiconductor materials might provide alternative opportunity for the morphology optimization. Fang et al. reported an inkjet printing assisted melt processing (IJP‐MP) method.^[^ ^48^ ^]^ In this method, ring‐like crystals of C8‐BTBT around the edge of the microtank patterns are formed after the inkjet printing and solvent drying. Then thermal annealing process above the melting point (102 °C) and slow cooling process were successively conducted, after which the C8‐BTBT molecules could fulfill the whole pattern with high crystallinity. Similarly, Jie et al. also managed to prepare C8‐BTBT arrays by conducting a dip‐coating and thermal annealing process on substrates with microchannels.^[^ ^49^ ^]^

#### 2.3.2 Meniscus‐Guided Coating

**Figure 6:**
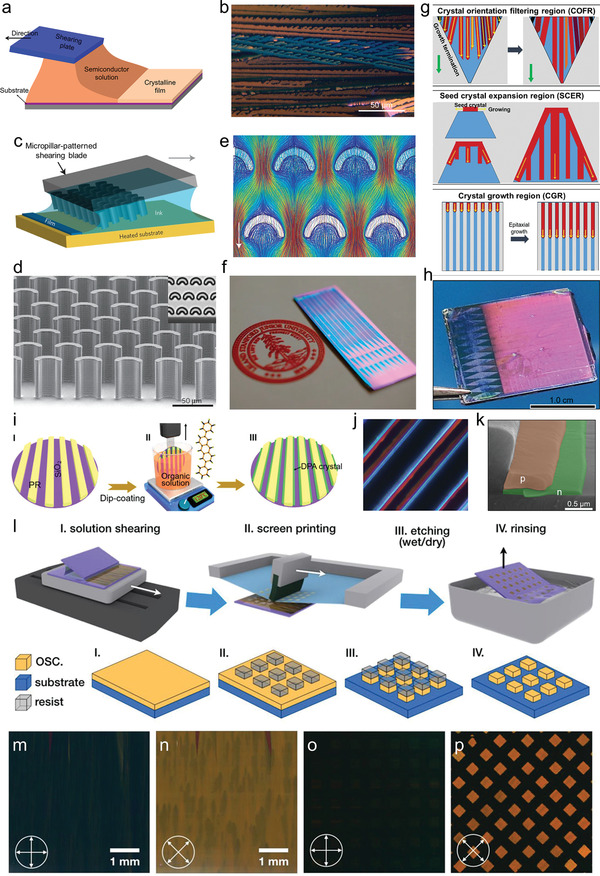
a) Schematic diagram of the solution‐shearing method. b) POM images of solution‐sheared TIPS‐PEN thin films. c) The schematic of solution shearing using a micropillar‐patterned blade. The arrow indicates the shearing direction. d) Scanning electron micrograph of a micropillar‐patterned blade. Inset, top view of the micropillars under an optical microscope. e) Streamline representation of simulated fluid flow around the micropillars. The arrow indicates the flow direction. f) A photographic image of one 1‐mm‐wide, 2‐cm‐long, single‐crystal OFET on a Si wafer. The blue regions are TIPS‐PEN single‐crystalline domains. Reproduced with permission.^[^ ^33^ ^]^ Copyright 2013, Springer Nature. g) Illustration of the “orientation filter funnel” concept. h) Photograph of a 1.5 × 1.5 cm^2^ C8‐BTBT OSSCF on the wafer. Reproduced with permission.^[^ ^34^ ^]^ Copyright 2022, Wiley‐VCH. i) Schematic illustration of the wafer‐scale growth of DPA crystal arrays by the CRMS method. Reproduced with permission.^[^ ^41^ ^]^ Copyright 2018, Elsevier. j) POM image of the bilayer p–n MB array acquired under the angle of 45. k) Corresponding cross‐sectional SEM image of the p–n bilayer MB. Reproduced with permission.^[^ ^56^ ^]^ Copyright 2019, ACS Publications. l) The schematic drawing of the procedures of solution‐processed printing and patterning of large‐area organic highly crystalline array. POM images of solution‐sheared highly crystalline film of C8‐BTBT: m) 0°, n) 45°. POM images of OSSC array of C8‐BTBT: o) 0°, p) 45°. The side length of the square shape in o) and p) is 150 µm. Reproduced with permission.^[^ ^57^ ^]^ Copyright 2020, Wiley‐VCH.

Meniscus‐guided coating of organic semiconductors presents a dramatic prospect for enabling low‐cost fabrication of large‐scale OSSC arrays. In the meniscus‐guided coating process, such as dip‐coating,^[^ ^50^, ^51^ ^]^ blade coating,^[^ ^52^ ^]^ bar coating,^[^ ^53^ ^]^ and solution shearing,^[^ ^54^ ^]^ a constant ambient force is applied by the unidirectional movement of the substrate or coating tool to trim the gas‐liquid‐substrate meniscus and realize the orientational OSSC growth. However, the high‐speed solvent evaporation rate, random nucleation and fluid flow instabilities in the coating process still hinder the large‐scale preparation of oriented OSSC arrays. For example, in the traditional method (**Figure** **6**a), voids and dendritic structures might exist in the OSSC arrays,^[^ ^55^ ^]^ and the orientation identity of the OSSC arrays cannot be guaranteed (Figure 6b). Modification of the coating process is needed to conquer the identical crystal orientation challenge, and optimization of the mass transport process should also be taken into consideration for high‐quality large‐scale OSSC growth.

In 2013, Bao et al. developed a fluid‐enhanced crystal engineering (FLUENCE) method.^[^ ^33^ ^]^ In this process, a series of triangular shapes followed by narrow slits were intentionally introduced to the solvent‐wetting regions, once the ink meniscus is formed at the triangle solvent‐wetting regions, nucleation is forced to start at the tip of the triangle, and the narrow slits could arrest undesired crystallites growth. Besides, shearing blade with micropillars was adopted to enhance the mass transport perpendicular to the coating direction to alleviate the dendritic structures. The shearing blade equipped with sub‐100‐µm crescent‐shaped micropillars (Figure 6c,d) could enhance lateral mass transport. As shown in Figure 6e, the arc of the micropillars separates the solvent flow and then generates recirculation behind the micropillars, which induces a lateral component in the flow. The narrow spacing between adjacent pillars induces rapid flow expansion, which accelerates the flow speed through the gap, further enhancing lateral mass transport, as evidenced by the fluid dynamic simulation as illustrated in Figure 6e, in which the lateral component is indicated using a white dashed‐line rectangle. The combined optimization of the nucleation and crystal growth process result in the realization of highly aligned centimeter‐sized 6,13‐bis(triisopropylsilylethynyl) pentacene (TIPS‐PEN) OSSC arrays (Figure 6f).

Similarly, Jie et al. reported an approach to achieve scalable solution printing of OSSC films with well‐aligned, uniformly orientated crystals by using an “orientation filter funnel” concept.^[^ ^34^ ^]^ In this setup, three distinct functional wetting/dewetting regions are engineered on the substrate: a V‐shaped crystal orientation filtering region (COFR), a triangular seed crystal expansion region (SCER), and a crystal growth region (CGR) with periodic wetting and dewetting striping patterns (Figure 6g). During the blade coating process, the funnel‐shaped solvent‐wetting COFR was first utilized to screen out the seed crystals with the same crystallographic orientation, followed by a triangle SCER for them to propagate laterally and longitudinally. Then, these uniaxial seed crystals continued their subsequent epitaxial growth at a CGR patterned with alternative wetting/dewetting lines, resulting in the formation of large‐area OSSC films (Figure 6g,h). The aligned C8‐BTBT ribbons have nearly identical widths and uniform thickness. A limitation of this strategy is the extremely long “runway” before the single crystal growth “takes off”, which may restrict its potential use in precision patterning. Except the elaborately designed triangular patterns to optimize the OSSC orientation identity, Jie et al. reported a strategy that combines microscale photoresist (PR) channels with the dip‐coating process, termed channel‐restricted meniscus self‐assembly (CRMS), for the wafer‐scale growth of 2,6‐diphenylanthracene (DPA) single‐crystal arrays with consistent crystal morphologies, qualities, and growth orientations.^[^ ^41^ ^]^ Dip‐coating technique features large meniscus front (usually at milli/centimeter‐size), bringing about heterogeneous nucleation behavior and polycrystalline molecular stacking during the crystal growth process. When the PR channels are immersed into organic solution, a contact line in the shape of a meniscus profile between two adjacent PR stripes will be formed due to surface tension effects (Figure 6i). Note that the meniscus is restricted within the PR channel due to the height of the PR stripes and the size of meniscus front diminishes to hundreds of nanometers. The preferential nucleation at the meniscus front near the PR and subsequent epitaxial growth would finally evolve into OSSC patterns. Furthermore, based on the similar principles and methods, they eventually realized a two‐component bilayer p‐n OSSC arrays by two dip‐coating in succession.^[^ ^56^ ^]^ Figure 6j,k shows the typical two types of micro‐belts partly overlap each other, TIPS‐PEN microbelts on the top of *N*,*N′*‐bis(2‐phenylethyl)‐perylene‐3,4:9,10‐tetracarboxylic diimide (BPE‐PTCDI) microbelts.

Despite the bottom‐up growth of OSSC arrays under the assistance of patterned structures, the etching of large‐scale highly crystalline films is an alternative selection. Based on the top‐down concept, Hu et al. managed to prepare centimeter‐scale C8‐BTBT OSSC arrays at centimeter scale (Figure 6l). First, they prepared a continuous highly crystalline C8‐BTBT thin film by solution shearing.^[^ ^57^ ^]^ Then aqueous resist polymer was screen printed on top of C8‐BTBT crystal, after etching of OSSCs and washing of photoresist, large‐scale C8‐BTBT OSSC arrays with highly ordered orientation were obtained, as evidenced by the simultaneous light extinction of OSSC arrays under POM (Figure 6m–p). Solution shearing method ensures the preparation of highly oriented crystal films with a thickness of a few molecular layers, so a significant advantage of this technique is that each individual pattern in the array originates from the same OSSC film, so the crystal quality, orientation and thickness are all the same.

In addition to the strategies mentioned above, Cho and coworkers developed an approach wherein Marangoni flow was induced using mixed solvents to overcome the coffee ring effect.^[^ ^58^ ^]^ In this process, first, the blend of crystalline small‐molecule semiconductor and an amorphous polymeric material would induce in vertical phase separation, which could improve the self‐stratified structure of OSSCs; secondly, pentagon shaped patterns with proper nucleation spot angles are adopted, which could promise highly aligned nucleation and single‐crystalline growth; third, solutal‐Marangoni effect is introduced by solvent additive which is crucial for the realization of uniform OSSC morphology. After the optimization of bar coating speed, pattern shape and substrate surface energy and solvent additives, highly aligned and highly ordered C8‐BTBT arrays are obtained.

**Figure 7:**
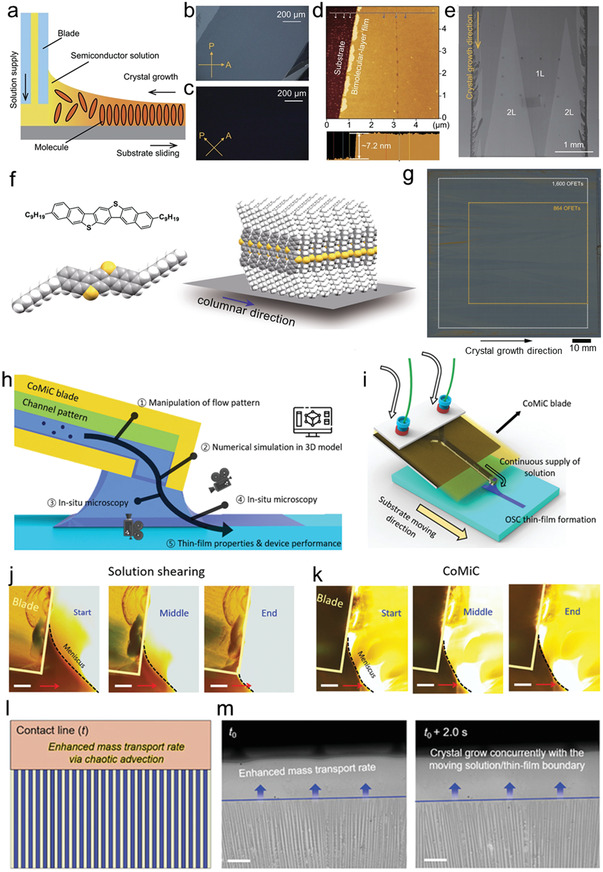
a) Schematic image of the continuous crystal growth method. b,c) POM images of the fabricated ultrathin C8‐DNBDT‐NW single crystals. d) AFM image and cross‐sectional profile of the 2L single crystal. e) Combined SEM image of ultrathin 1L and 2L single‐crystalline films. Reproduced with permission.^[^ ^19^ ^]^ Copyright 2018, AAAS. f) Molecular structure of C9‐DNBDT‐NW and schematic image of the structure of the molecular assembly. g) Confocal microscopy image of 90 mm by 90 mm C9‐DNBDT‐NW crystal on silicon wafer substrate. The direction of shearing, corresponding to the crystal growth direction, was from left to right. Reproduced with permission.^[^ ^20^ ^]^ Copyright 2019, Springer Nature. h) Schematic diagram of CoMiC‐based comprehensive analytical system along the entire flow path connecting flow pattern, crystallization, and thin‐film properties. i) Schematic diagram of continuous supply of solution via microfluidic chip. j, k) Side‐view in situ image analysis of meniscus shape variation during the coating of doped TIPS‐PEN thin‐film transistors fabricated by conventional solution shearing and CoMiC without 3D structures. The red arrows indicate substrate moving direction and the scale bars are 30 µm. l,m) Situ microscopy images and schematic illustration showing the variation of solution/thin‐film boundary and crystallization process of doped TIPS‐PEN using the continuous‐flow microfluidic‐channel‐based meniscus‐guided staggered herringbone microfluidic channel coating (SHM‐CoMiC). Reproduced with permission.^[^ ^59^ ^]^ Copyright 2020, Wiley‐VCH.

Despite modification of substrate or coating tools, another concern of growing large‐scale crystalline thin films is the solvent supply. A constant incoming solvent flow rate precisely matching the evaporation rate guarantees the mass conservation during the process and is important to maintain a stable meniscus profile. A meniscus‐guided solution process, named continuous edge casting, fulfills this requirement and demonstrates the ability for wafer‐scale organic crystalline thin film deposition.^[^ ^19^ ^]^ **Figure** **7**a illustrates the experimental setup of the equipment, and 3,11‐dioctyldinaphtho[2,3‐d:20,30‐d0]benzo[1,2‐b:4,5‐b0]dithiophene (C8‐DNBDT‐NW) is used as a benchmark material. A rectangular blade is placed near the substrate, creating a straight three‐phase contact line parallel to the blade edge, which is helpful to grow a large‐size crystal domain (Figure 7b–d). Meanwhile, the organic semiconductor solution is supplied from inside of the blade at the same rate as the solvent evaporation. Crystalline films of centimeter‐long and thickness confined at 1–2 molecular layers are obtained by precisely control the solution concentration, substrate moving speed, and substrate temperature, as shown in the SEM image in Figure 7e. Then a dry‐etching process was conducted to pattern the large‐scale crystalline film. Furthermore, 3,11‐dinonyldinaphtho[2,3‐d:2′,3′‐d’]benzo[1,2‐b:4,5‐b’]dithiophene (C9‐DNBDT‐NW) was also applied for meniscus‐driven printing. Compared to the previous alkyl‐modified DNBDT derivatives, C9‐DNBDT‐NW is probably the organic semiconductors with the best manufacturability because of its high solubility (Figure 7f). Based on the above considerations and the previous fabrication process, they further optimized the growth parameters of the film in detail and produced a 90 mm × 90 mm wafer‐level C9‐DNBDT‐NW OSSC on a silicon wafer substrate (Figure 7g).^[^ ^20^ ^]^ Another example of continuously providing solution is provided by Lee et al. They developed a continuous‐flow microfluidic‐channel‐based meniscus‐guided coating (CoMiC) process to manipulating the flow patterns (Figure 7h,i).^[^ ^59^ ^]^ Continuous supply of a solution of an organic semiconductor with various flow patterns is generated using microfluidic channels. One of the features of CoMiC is the continuous supply of solution during coating, which is on the contrary to conventional solution shearing (Figure 7j), where a fixed amount of solution is inserted between the blade and the substrate prior to thin film coating. Figure 7k is side‐view high‐resolution in situ images of the meniscus during CoMiC (here, the microfluidic channel did not have any 3D structuring). For CoMiC, shape of the meniscus remains the same during coating due to the maintenance of steady state, where the evaporating solvent and depositing thin‐film are replenished by the constant supply of solution. Surprisingly, in the case of staggered herringbone microfluidic channel coating (SHM‐CoMiC), the “lag behind/catch up process” did not occur; ribbons in all regions grew concurrently with the moving solution/thin‐film boundary (Figure 7l,m). The continuous supply of a solution guarantees steady meniscus shape, which is beneficial for preparing large scale oriented OSSCs.

#### 2.3.3 Liquid Bridge‐Mediated Patterning

Capillary bridges are commonly found in life and in nature as a minimized region of liquid between the surfaces of two arbitrarily sized rigid objects (usually solids, but in rare cases liquids as well). For a capillary liquid bridge between two parallel planes, capillary force and surface tension are the key driving forces controlling its shape and infiltration or de‐infiltration behavior. Capillary bridges can regulate the transport direction of the capillary flow, thus suppressing uncontrollable de‐infiltration processes such as the coffee ring effect and the Marangoni effect.

**Figure 8:**
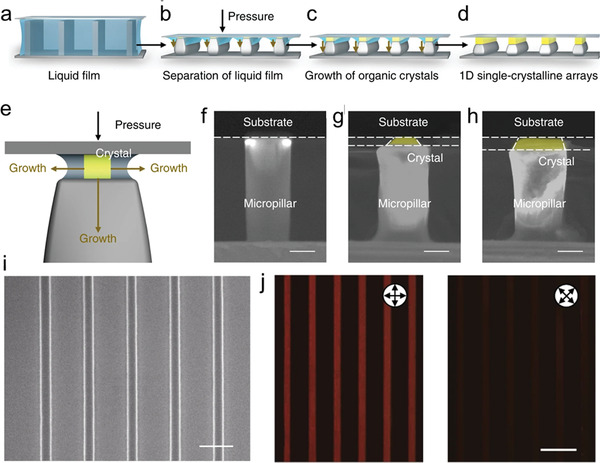
a–d) Schematic illustration of 1D organic arrays assembly processing under pressure. e) Zoom‐in schematic illustration of 1D organic crystal nucleus growing in the confined space. f–h) SEM images with cross‐sectional view of organic nanostructure growing on an individual micropillar, scale bars: 3 µm. i) SEM image of 1D organic single‐crystal arrays with precise alignment and regular morphology. j) Parallel‐polarized and cross‐polarized reflection images of 1D arrays. Reproduced with permission.^[^ ^60^ ^]^ Copyright 2019, Springer Nature.

The liquid‐bridge has been applied for transferring OSSC arrays grown in a template to target rigid or flexible substrates.^[^ ^46^ ^]^ Besides, liquid‐bridge could be applied directly for OSSC growth.^[^ ^60^, ^61^, ^62^, ^63^ ^]^ Wu et al. utilized a micropillar‐structured PDMS template, and the organic‐semiconductor solution was carefully dropped on it, then a target flat substrate was covered on the solution. The process of dewetting of organic solutions began with the confinement of the liquid in the spaces of the microstructured pillars. As the solvent gradually evaporates, the continuous liquid film breaks into discrete capillary bridges anchored at the top of the micropillars. Further solvent evaporation induced in the shrink of capillary bridges and crystallization of organic molecules at the capillary bridges. After complete evaporation of the solvent, regulated OSSC arrays were finally obtained on the target substrates (**Figure** **8**a–d). The thickness of the confined space is regulated by the autogenous evolution of the liquid volume under the applied tunable pressure (Figure 8e–j). As a result, OSSC arrays with sequentially tunable heights ranging from less than 10 nm to ≈1 µm are controllably obtained. This technique provides excellent universality for diverse types of materials and substrates. However, the preparation of the templates would involve a more complicated lithography process.

#### 2.3.4 Triple‐Phase Contact Line Regulation Assisted Patterning

Triple‐phase contact line (TPCL) is the specific location where nucleation mainly occurs attributed to the relatively high evaporation rate of solvent near it. It has been proved that proper regulation of TPCL would effectively tunning the nucleation.

##### Liquid‐Substrate OSSC Growth

**Figure 9:**
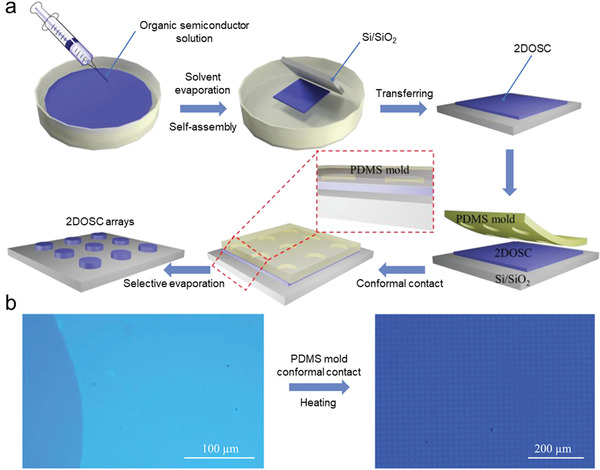
a) Schematic of patterning high‐resolution layer‐controlled 2D OSSC arrays. b) OM images of large‐area C6‐DPA 2DOSSC and high‐resolution 2D OSSC arrays. Reproduced with permission.^[^ ^64^ ^]^ Copyright 2021, Wiley‐VCH.

Hu group reported a two‐step strategy for patterning high‐resolution layer controlled of 2D OSSCs (2D OSSCs).^[^ ^64^ ^]^ First, 2D OSSCs with a well‐defined number of layers were obtained on a large scale by a solution‐treated organic semiconductor crystal engineering method; then, high‐resolution arrays of 2DOSSCs with a controlled number of layers were fabricated by a PDMS template‐assisted selective contact evaporation printing technique (**Figure** **9**a,b). An obvious virtue of this process is that every individual pattern in the array is derived from the same 2,6‐bis(4‐hexylphenyl)anthracene (C6‐DPA) OSSC film prepared by solution‐treated crystal engineering, so the crystal quality, orientation and thickness are identical. Another crucial feature is that the subsequent patterning does not involve solution process.

##### Droplet‐Pinned Crystallization (DPC) Method

**Figure 10:**
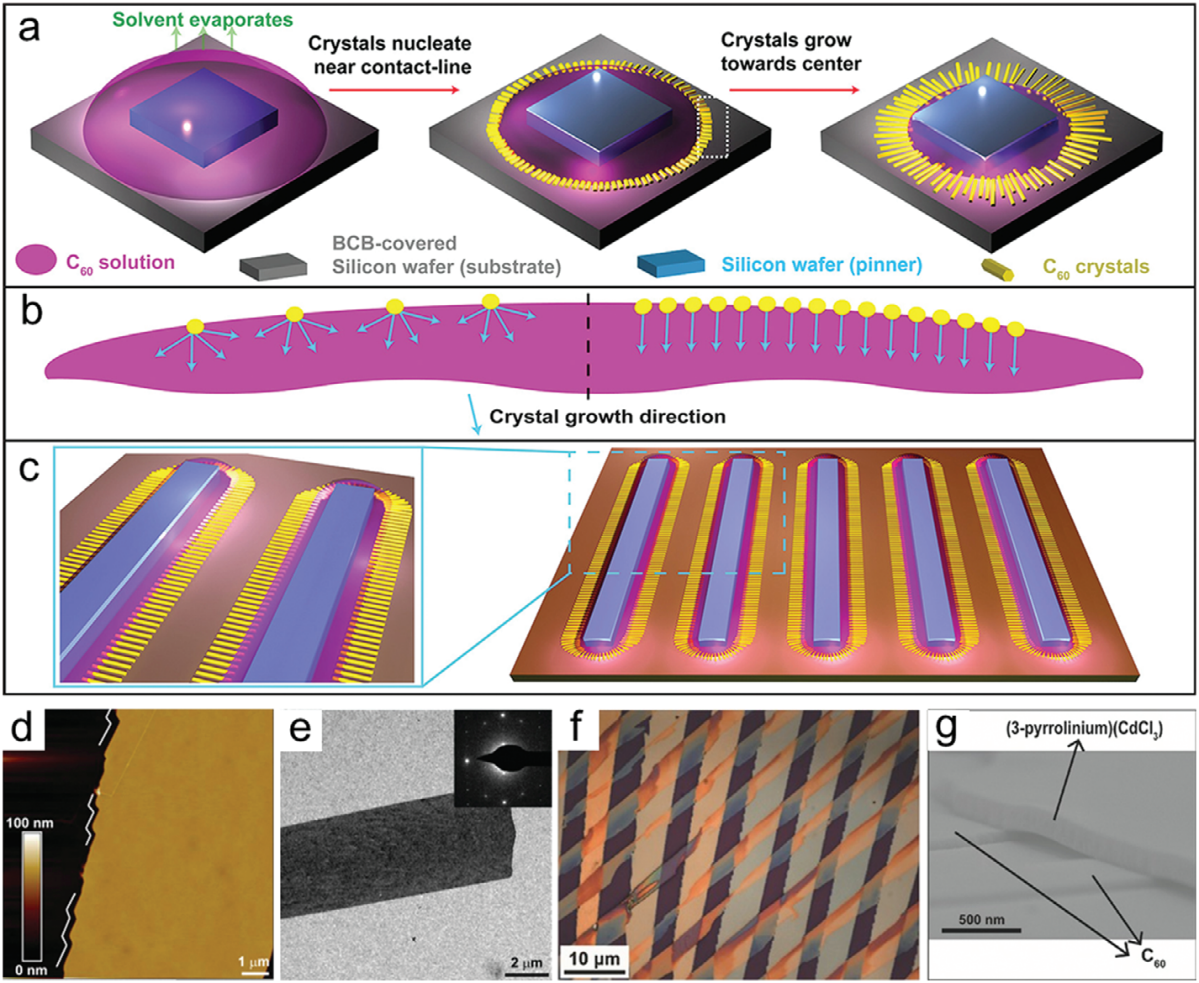
a) Schematic representations of the DPC method. b) A magnified view of a white‐marked area in (a). c) DPC can be scaled up by using multiple pinners with larger sizes. d) AFM image of a ribbon crystal. White lines highlight the faceted edges. Height of 10 ribbon crystals was measured, giving an average of 57 ± 7 nm (SD). e) Transmission electron microscopy (TEM) image of a ribbon; (inset) selected‐area electron diffraction (SAED) pattern containing a single set of spots, indicating the single crystallinity of the ribbon. SAED at different locations of the ribbon showed identical patterns. Reproduced with permission.^[^ ^65^ ^]^ Copyright 2012, ACS Publications. f) The AFM of bilayered heterojunctions. g) A SEM image (side view) of the bilayered heterojunctions. Reproduced with permission.^[^ ^66^ ^]^ Copyright 2015, Wiley‐VCH.

In the traditional drop‐casting method, the recession of the TPCL of the solution on the substrate is irregular, leading to unsteady, nonuniform deposition. In order to achieve aligned OSSC arrays, Bao et al. developed a droplet‐pinned crystallization (DPC) method by adjusting TPCL pinning to control the nucleation site and growth direction.^[^ ^65^ ^]^ In this process, they placed a pinner on top of a substrate, then drop‐casting is carried out (**Figure** **10**a,b). During drying of a droplet, crystals nucleate near the TPCL and grow along the receding direction (toward the pinner in the center) of the droplet and finally evolve into large crystals (Figure 10c). Based on this method, large area OSSC arrays of C60 were obtained (Figure 10d,e). By successively depositing materials in orthogonal solvents, for example C60 in mixed solvents of m‐xylene and carbon tetrachloride and (3‐pyrrolinium) (CdCl_3_) in ethanol, bilayer single‐crystalline ribbons of CdCl_3_ grown on top of well‐aligned C60 ribbons were obtained (Figure 10f,g).^[^ ^66^ ^]^ However, because of the lack of effective confinement of the template, the orientation and size of crystals obtained by this method are inconsistent and the angle between the bilayer structures might vary from batch to batch.

##### Liquid‐Atmosphere Boundary Regulating

**Figure 11:**
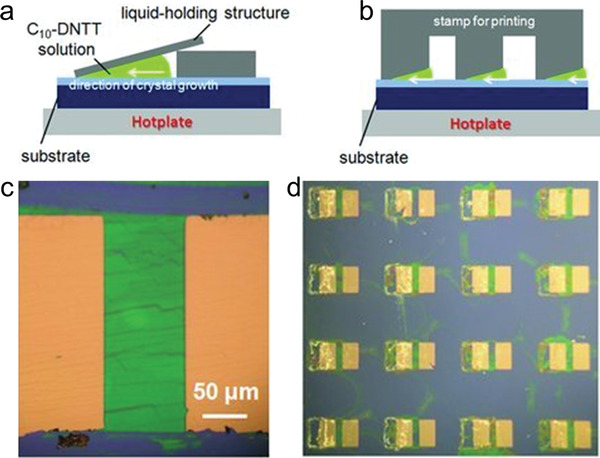
a) Method for preparing individual C10‐DNTT organic transistors. b) Method for fabricating the C10‐DNTT transistor arrays. c) An OM image of the individually grown film. d) An OM image of the grown film arrays. Reproduced with permission.^[^ ^67^ ^]^ Copyright 2011, Wiley‐VCH.

Besides, another static meniscus guides were employed during solvent evaporation: edge casting. In the edge casting technique, the meniscus is guided by the inclined surfaces that cover the solution droplet during solvent evaporation (**Figure** **11**a–d). Due to the high local concentration of molecules near the liquid‐gas boundary on the open side, the crystalline film of 2,9‐alkyldinaphtho[2,3‐b:20,30‐f]thieno[3,2‐b]thiophene (C10‐DNTT) grows with the gradual movement of the boundary toward the closed edge. With this method, the authors exhibited aligned crystal arrays of C10‐DNTT.^[^ ^67^ ^]^ Patterned deposition over large regions has also been realized in this work.

##### Other Strategies to Regulate Triple‐Phase Contact Line

**Figure 12:**
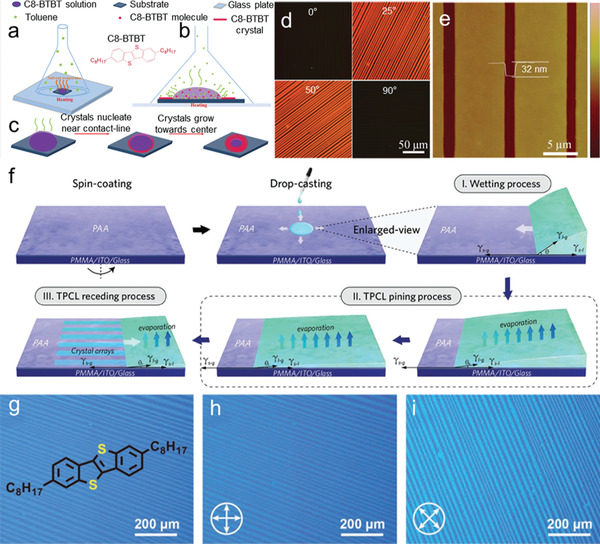
a) The substrate was placed on a hot plate and covered with a funnel, through which the solvent evaporates. b) Evaporation of solvent results in appropriate vapor pressure in this semiclosed space. c) As the solvent evaporates quickly through heating, C8‐BTBT molecules nucleate near the contact line of the droplet. Subsequently, the nuclei grow along the receding direction (toward the center) of the droplet orderly under the solvent vapor. d) POM images of C8‐BTBT crystals on briefly washed SiO_2_/Si. e) AFM image of C8‐BTBT ribbon crystals on benzocyclobutene (BCB). Reproduced with permission.^[^ ^68^ ^]^ Copyright 2018, RSC publishing. f) Schematic illustration of the fabrication procedure of solution‐processed large‐area aligned C8‐BTBT single crystals on the PAA surface. g) Optical images of C8‐BTBT single‐crystal arrays. h,i) POM images of aligned C8‐BTBT single crystals. Reproduced with permission.^[^ ^69^ ^]^ Copyright 2022, Wiley‐VCH.

It is known that, the nucleation of organic semiconductors would change the dewetting behavior of the TPCL, since the wetting properties created by the newly formed TPCL (gas/solution/nucleus) might be different from the pristine TPCL (gas/solution/substrates). To balance these parameters, Jiang et al. developed a limited solvent vapor‐assisted crystallization (LSVC) method to enable steadily withdraw of the TPCL.^[^ ^68^ ^]^ In this process, a semienclosed space is established using an up‐side‐down funnel (**Figure** **12**a), which contributes to maintaining a favorable solvent vapor pressure to prevent abrupt precipitation and offers enough time for mass transport, thus facilitating the preferred alignment of nuclei growth toward the droplet receding direction (Figure 12b,c). The similar wetting behaviors of toluene on substrates and C8‐BTBT crystals further guarantee a steady TPCL under vapor evaporation. Based on the LSVC method, highly ordered C8‐BTBT crystal arrays were obtained (Figure 12d,e). Though no expensive equipment is needed in this method, however, the universality and large‐scale production of the OSSC arrays need further improvement.

Furthermore, Hu group report a poly (amic acid) (PAA) modified substrates as a growth template to produce highly aligned OSSCs over a large area using a simple drop‐casting method.^[^ ^69^ ^]^ PAA can be applied as a surface‐assisted crystallization template due to three critical features: 1) the high surface energy guarantees complete spreading of the solution; 2) its distinctive surface characteristics enable the formation of stable TPCL when the solution evaporates, similar to the meniscus‐induced growth technology; 3) the unique nanogrooves on the surface make it efficient to induce molecular crystallization. The process of assembling large‐area C8‐BTBT single‐crystal arrays on the PAA surface is divided into three main steps, including wetting, three‐phase line pinning and three‐phase line receding (Figure 12f). The OM and POM image of the prepared C8‐BTBT OSSCs displays microstripe array morphology and homogeneous color over the entire substrate as shown in Figure 12g,i. This promising feature enables large‐area assembly of ultrathin, highly aligned single‐crystal arrays for a variety of organic semiconductor molecules, demonstrating good applicability.

### 2.4 Direct Construction of Device Arrays

**Figure 13:**
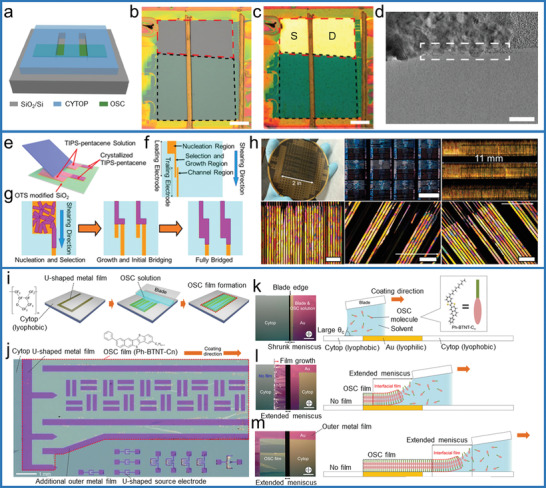
a) Schematic of CYTOP printed on C6‐DPA monolayer crystalline thin film. b) OM image of printed CYTOP. The area in black dotted polygon is monolayer crystalline film, in red dotted polygon is exposed substrate, and others are printed CYTOP. c) Silver source/drain electrodes from silver mirror reaction. d) Cross‐sectional TEM image showing the overlapping region of electrode and crystalline film. Reproduced with permission.^[^ ^70^ ^]^ Copyright 2021, Springer Nature. e) Schematic depiction of film growth during solution shearing. f) Depiction of our electrode design with labeling of different regions. g) Description of CONNECT. h) Optical characterization of patterned TIPS‐PEN crystalline domains. Reproduced with permission.^[^ ^7^ ^]^ Copyright 2015, PNAS. i) Schematic of the EMG coating technique. j) OM of printed Ph‐BTNT‐Cn films fabricated on CYTOP surfaces with gold films predefined for the EMG coating. Photo credits: Gyo Kitahara, University of Tokyo. k–m) Shape of solution meniscus during the coating. Reproduced with permission.^[^ ^39^ ^]^ Copyright 2020, AAAS.

To obtain patterned devices based on the above mentioned OSSC arrays, shadow mask assisted metal evaporation process is followed to form top‐contact electrodes on OSSCs. Compared with the high energy consumption and high temperature process of vacuum evaporation process, construction of top‐contact electrodes pairs in relatively mild condition is of great significance, especially in ultra‐thin OSSC devices. In 2022, Jiang et al. realized this goal through the combination of hydrophobic/hydrophilic pattern and silver mirror reaction (SMR).^[^ ^70^ ^]^ This process causes little damage to ultrathin crystals and could output large scale arrays. In this setup, monolayer films were prepared by drop‐casting on hydrophilic substrate, and hydrophobic/hydrophilic patterns on monolayer films were realized by printing of a CYTOP layer with pierced area on top (**Figure** **13**a,b), the exposed hydrophilic substrate and printed CYTOP forms the pattern. The SMR results in the formation of conductive silver films and the film could slightly epitaxial into crystalline films forming source/drain contacts, with length of about 250 nm (Figure 13c,d).

Besides top‐contact electrodes, crystals could also be prepared at substrates with patterned electrodes applying similar techniques addressed in Section 2.2. For example, Bao et al. achieved patterned growth of several kinds of OSSC OFET arrays via selectively modifying OTS around the prepatterned Au electrode pads based on the hydrophilic/hydrophobic pattern and dip‐coating process.^[^ ^71^ ^]^ A similar procedure was adopted by Kim et al, after ink‐jet printing, the better wetting ability of solution on Au electrodes would induce in nucleation and growth of TIPS‐PEN crystals from Au electrodes and gradually crossing the hydrophobic OTS modified channel area.^[^ ^72^ ^]^

It is known that patterns with elaborately designed structures could guide the crystal orientation or solution spreading behaviors.^[^ ^33^, ^34^ ^]^ Thus, electrodes with similar functions could also be applied to improve the orientation of OSSCs. For instance, Park et al. introduce a method, “controlled OSSC nucleation and extension for circuits (CONNECT),” that uses differential surface wetting properties combined with solution shearing to pattern and align crystalline domains of OSSCs selectively between source and drain electrodes in a single step.^[^ ^7^ ^]^ In the CONNECT process (Figure 13e–g), the nucleation happens at the leading electrodes, the trailing electrode would facilitate the selection and growth of oriented OSSCs. The OSSCs only crystallize on the solvent wetting electrodes and across the channel, effectively creating self‐patterned and precisely registered crystalline domains across the channel. This method ensures that each bridging event yields aligned crystalline domains from a single nucleation site across the channel (Figure 13h).

The spreading of semiconductor solution and growth of OSSCs on lyophobic surface is limited and inefficient, however, the growth of OSSCs on lyophobic dielectrics, such as CYTOP, would obtain low trap density and thus high performance. In light of this, Hasegawa et al. developed an extended meniscus‐guided (EMG) coating technique that allows to manufacture OSSC films on top of highly lyophobic CYTOP gate dielectric layer.^[^ ^39^ ^]^ In situ microscope observations were conducted for the blade coating on the CYTOP surface with U‐shaped metal film patterns (Figure 13i,j). Before the receding edge of the solution meniscus reached the metal film area, the solution meniscus was strongly confined under the blade with a large *θ*_c_, as seen from a relatively narrow width of dark vertical line area in Figure 13k. The feature is due to the highly lyophobic nature of the CYTOP surface, where no OSSC film was obtained. When the receding edge of the solution meniscus reached the bottom of the U‐shaped metal film area, the meniscus began to be extended along the receding direction, as seen from an increasing width of the dark area in Figure 13l. After the fairly thin solution layer was formed at the receding edge on the metal film surface, uniform OSSC film growth began. The eventual extended meniscus on the CYTOP surface was accompanied by a successive OSSC film growth, as presented in Figure 13m. The obtained large‐scale crystal film has defined thickness of bi‐molecular layers, which is highly related to the preferred growth mode of phenyl/alkyl‐substituted benzothieno[3,2‐b]naphtho[2,3‐b]thiophene (Ph‐BTNT‐Cn) and the application of bi‐component semiconductors with different alkyl chain length.

## 3 Patterning of MMCs

The emerging MMCs in recent years offered a novel platform for the construction of OSSC arrays with uniform thickness. MMCs related study has achieved substantial improvement since Hu and co‐workers successfully prepared monolayer OSSCs in 2011.^[^ ^73^ ^]^ Decrease of the bulk crystal thickness down to a few layers or even monolayer holds tremendous possibilities for the production of high‐performance transistors that may demonstrate unprecedented optoelectronic behavior.^[^ ^73^, ^74^, ^75^, ^76^, ^77^ ^]^ For instance, the mobility of MMC OFETs is on par with their bulk counterparts, and the lowered contact resistance in MMC OFETs enables them to be superior in short channel and low power devices.^[^ ^74^, ^75^ ^]^ Besides, MMCs would eliminate the device‐to‐device performance fluctuation due to their identical access resistance and long‐range ordered molecular packing, which is critical to obtain uniform device arrays. Moreover, the direct exposure of the conductive channel provides MMC OFET with superior sensing features^[^ ^76^ ^]^ and so on. Another thing that cannot be ignored is the monolayer properties of MMCs will spread very well on the various substrates because of their excellent flexibility at thickness limit.^[^ ^77^ ^]^ All the features mentioned above make MMCs very attractive for constructing device arrays with uniform performances.

**Figure 14:**
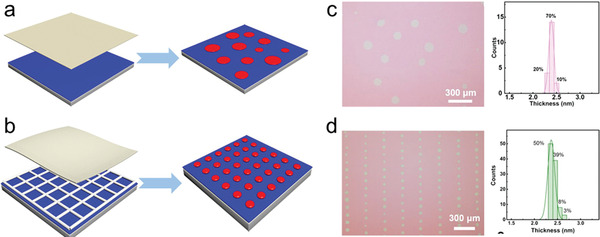
a) The schematic diagram of STAA method (red: C6‐DPA MMCs; blue: SiO_2_; gray: Si^++^; yellow: filter paper; green: C6‐DPA solution). b) The schematic diagram of preparing MMC patterns by STAA method with dual template (yellow: filter paper; white: metal mesh; blue: SiO_2_; gray: Si^++^). c) OM images of C6‐DPA MMCs fabricated by STAA method on the SiO_2_/Si^++^ substrate and the corresponding histogram of thickness distribution of 10 C6‐DPA MMC plates. d) OM image of C6‐DPA MMC arrays fabricated by STAA method with dual template and the corresponding histogram of thickness of 100 C6‐DPA MMC plates. Reproduced with permission.^[^ ^35^ ^]^ Copyright 2022, Wiley‐VCH.

Similar to the nanostructured templates induced patterning of OSSCs, Jiang et al. presented a soft‐template‐assisted assembly (STAA) method to prepare MMCs with uniform morphology over millimeter scale by capillary restricted‐domain crystallization.^[^ ^35^ ^]^ The periodic arrays were obtained by periodic contact between the bottom metal mesh template and substrates and the thickness is controlled by the steady capillary force generated by the top soft templates with absorbing ability and slowing down the solvent evaporation. By regulating the mesh density of the rigid template, several MMC arrays with tunable resolution and millimeter‐scale were obtained (**Figure** **14**a–d). The universality of the method was proved by a series of small‐molecule semiconductors. Though thickness is highly controlled, however, the orientations of the MMCs are not identical.

## 4 OFET and Device Applications of OSSC Arrays

### 4.1 OFET Performance

**Figure 15:**
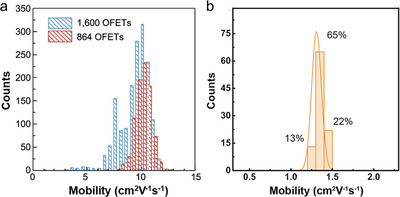
a) Statistics for measured mobility of 1600 C9‐DNBDT‐NW OFETs from a 40 × 40 array. Reproduced with permission.^[^ ^20^ ^]^ Copyright 2019, Springer Nature. b) Histograms of saturation mobility of 100 C6‐DPA OFETs from a 10 × 10 array. Reproduced with permission.^[^ ^35^ ^]^ Copyright 2022, Wiley‐VCH.

**Table** **1** shows the summary of the OFET mobilities of above‐mentioned typical arrays, the OFET device performance fluctuation has achieved enormous improvement with the CV value, but the limitations on large‐scale device arrays remain to be explored and settled. Amongst them, the most attractive CV value‐related works are shown in **Figure** **15**. The CV of central 864 C9‐DNBDT‐NW OFETs is 7.2% of the 90 mm × 90 mm wafer‐level OSSC on a silicon wafer substrate. However, For the entire 1600 OFETs, the CV value is slightly larger, which might be caused by the uneven film thickness at the edges of substrate.^[^ ^20^ ^]^ Besides, the smallest CV obtained for C6‐DPA OFET arrays is partially induced by the identical thickness of the 100 crystal arrays.^[^ ^35^ ^]^ It is essential to be aware that the orientation is not identical for all the crystals, but the narrow distribution inspired us to investigate the anisotropic charge transport in C6‐DPA MMC.

**Table 1:** Summary of OFET array electrical performances

Materials	Method	Max mobility	Avg. mobility	CV	Ref
Rubrene	PVT	2.4 cm^2^ V^−1^ s^−1^	/	/	[40]
C8‐BTBT	Inkjet printing	31.3 cm^2^ V^−1^ s^−1^	16.4 cm^2^ V^−1^ s^−1^	/	[18]
TIPS‐PEN	Inkjet‐assisted nanotransfer printing	/	1.45 cm^2^ V^−1^ s^−1^	/	[46]
TIPS‐PEN	Solution coating	11 cm^2^ V^−1^ s^−1^	8.1 cm^2^ V^−1^ s^−1^	14.8%	[33]
C8‐BTBT	Orientation filter funnel	/	8.30 cm^2^ V^−1^ s^−1^	9.8%	[34]
DPA	Channel‐restricted meniscus self‐assembly	39.3 cm^2^ V^−1^ s^−1^	30.3 cm^2^ V^−1^ s^−1^	17.6%	[41]
C10‐DNTT	3D template limit	11 cm^2^ V^−1^ s^−1^	7 cm^2^ V^−1^ s^−1^	/	[67]
C8‐BTBT	Inkjet printing	9.33 cm^2^ V^−1^ s^−1^	6.31 cm^2^ V^−1^ s^−1^	/	[48]
C8‐BTBT	Gap‐controlled bar coating	20.6 cm^2^ V^−1^ s^−1^	/	/	[58]
C8‐DNBDT‐NW	Continuous edge casting	13 cm^2^ V^−1^ s^−1^	/	/	[19]
C6‐DPA	Soft‐template‐assisted assembly	/	1.3 cm^2^ V^−1^ s^−1^	4.4%	[35]
C_60_	Droplet‐pinned crystallization	11 cm^2^ V^−1^ s^−1^	5.2 cm^2^ V^−1^ s^−1^	40.4%	[65]
C8‐BTBT	PAA surface‐assisted assembly	18.6 cm^2^ V^−1^ s^−1^	15.9 cm^2^ V^−1^ s^−1^	/	[69]
C6‐DPA	Selective contact evaporation printing	1.9 cm^2^ V^−1^ s^−1^	1.6 cm^2^ V^−1^ s^−1^	12.5%	[64]
C8‐BTBT	Limited solvent vapor‐assisted crystallization	6.2 cm^2^ V^−1^ s^−1^	4.2 cm^2^ V^−1^ s^−1^	/	[68]
C8‐BTBT	Solution shearing	8.7 cm^2^ V^−1^ s^−1^	6.4 cm^2^ V^−1^ s^−1^	14%	[57]
TIPS‐PEN	Solution shearing	0.4 cm^2^ V^−1^ s^−1^	0.17 cm^2^ V^−1^ s^−1^	28%	[7]
Ph‐BTNT‐Cn	Meniscus guided coating	5.5 cm^2^ V^−1^ s^−1^	/	/	[39]
C9‐DNBDT‐NW	Continuous edge‐casting	/	10.1 cm^2^ V^−1^ s^−1^	7.2%	[20]
TIPS‐PEN	CoMiC	/	2.04 cm^2^ V^−1^ s^−1^	7.3%	[59]

**Figure 16:**
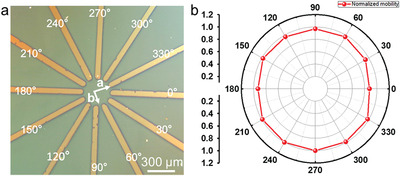
a) POM images of polymer‐assisted transferring patterned fan shaped electrodes on millimeter scale C6‐DPA MMC. b) Radar graph of normalized mobilities on different directions of C6‐DPA MMC. Reproduced with permission.^[^ ^78^ ^]^ Copyright 2022, RSC publishing.

In light of this, we developed a simple and nondestructive top‐electrode dry transferring method (**Figure** **16**a). We found that the C6‐DPA MMCs exhibit a low mobility anisotropy ratio of ≈1.15 (Figure 16b).^[^ ^78^ ^]^ It is worth highlighting that the weak mobility anisotropy of C6‐DPA MMCs is also essential for the narrow performance distribution.

### 4.2 Device Applications

As an emerging research field, OSSC electronics has been extensively pursued. The applications of OSSCs in diodes, transistors, functional devices (such as phototransistors, memory devices, sensors and detectors), and integrated devices or circuits have been intensively studied. In the integrated device application, minimal crosstalk between the adjacent devices and outstanding device reliability and reproducibility are prerequisites. In the above‐mentioned section, we have extensively summarized the widely available techniques used for accurate patterning of OSSC. With these approaches, large‐scale OSSC‐pattern based OFET arrays have been prepared. The encouraging achievements of high gain inverters, human electrocardiogram signals detection, bionic vision, image sensors and high‐speed rectifier are highlighted in this section, showing a promising future of the OSSC in integrated circuits.

#### 4.2.1 Image Sensing

**Figure 17:**
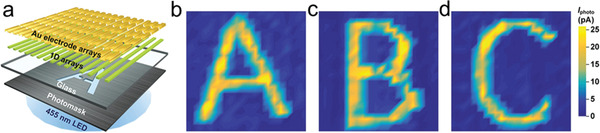
a) Schematic illustration of the image sensor based on 1D array photodetectors. b–d) False‐color 2D photocurrent mappings of image sensors, illustrating the detection of optical images of “A,” “B,” and “C.” Scale bar, 200 µm. Reproduced with permission.^[^ ^63^ ^]^ Copyright 2021, Wiley‐VCH.

Organic image sensors will be intriguing owing to possibly extended spectrum, mechanical flexibility, low cost, and scalable production. **Figure** **17**a presents the schematic diagram of the image sensor based on organic 1D array photodetectors. The main elements of the imaging device include a 455 nm blue LED, a stainless‐steel patterned photomask, a glass substrate, and organic 1D array photodetectors. Based on the scalable fabrication of 1D array photodetectors, 20 × 20 multiplexed image sensors with high accuracy are demonstrated by Qiu et al. for capturing the light signals of capital letter “A,” “B,” and “C” (Figure 17a–d).^[^ ^63^ ^]^

#### 4.2.2 Bionic Visual Perception

**Figure 18:**
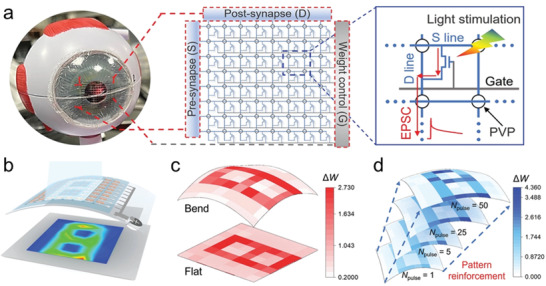
a) Image of the flexible FSP‐OFET array attached to the human eyeball model and detailed photosynaptic array design. b) Schematic of the measurement process. c) Measured results of a number “8” pattern under flat and bending states, respectively. d) Measured results of a number “8” pattern in the initial state and after pulse numbers of 1, 5, 25, and 50, respectively. Reproduced with permission.^[^ ^79^ ^]^ Copyright 2022, Wiley‐VCH.

Vision, as an effective means of acquiring information, is recognized as one of the most essential senses for living organisms. Based on the properties of the vision generation process, it is suggested that solid‐state devices with both light‐sensing and synaptic functions are supposed to be a prerequisite for the achievement of artificial vision systems. Bionic vision and image learning has been realized by Zhang et al. using the device arrays (**Figure** **18**a–d).^[^ ^79^ ^]^ They collected the change in current at each pixel under multiple light exposures, and as the number of light exposures increased, more powerful photocurrents resulted in higher image contrast. This process is similar to the process by which the human eye slowly remembers objects by looking at the same thing repeatedly. Combined with the recognition of objects by computerized convolutional neural networks (CNNs), it can be observed that the recognition ability of CNNs for images enhances as the number of light illuminations increases, which strongly proves the potential of fully solution‐printed photosynaptic (FSP) transistor arrays for applications in the field of bionic vision.

#### 4.2.3 Bioelectronic Sensing

**Figure 19:**
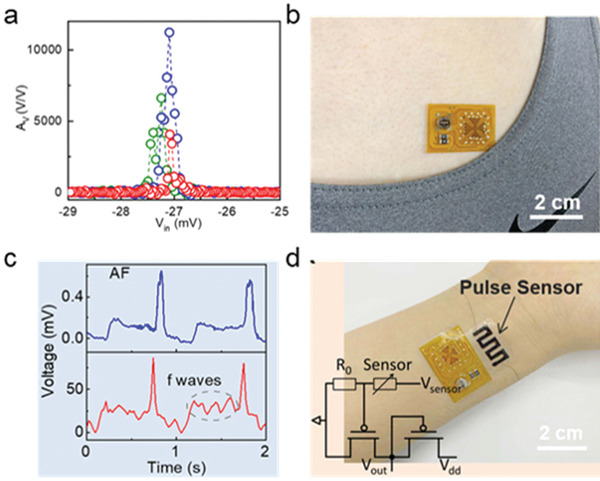
a) Av as a function of input voltage derived from voltage transfer characteristics under *V*_dd_ = −1, −2, and −3 V, respectively. b) Photograph of a flexible amplifier module attached to a human chest. c) Unconditioned and amplified pulse signal taken on the same human subject. d) Photograph of an integrated pulse sensor using flexible carbon nanotube films and amplifier circuit. Inset shows the circuit diagram. Reproduced with permission.^[^ ^80^ ^]^ Copyright 2022, Springer Nature.

Cardiovascular diseases have been one of the leading causes of death worldwide. Many patients probably do not be aware of their diseases. Therefore, wearable continuous electrocardiogram (ECG) monitoring equipment will be a pivotal tool in saving valuable lives. The key problem faced by wearable electronic devices is the need to achieve flexibility, low power consumption and amplification of weak signals at the same time. As a classical example, Wang et al.^[^ ^80^ ^]^ successfully fabricated inverters and obtained voltage gain of 1.1 × 10^4^ at 3 V (**Figure** **19**a), which is the highest value reported so far. The low operating voltage enables the fabrication of a coin cell battery‐powered flexible amplifier chip and application in detection and amplification of human electrocardiogram (ECG) signals. The chip can amplify human ECG signals more than 300 times and maintain high fidelity, and its ability to detect weak ECG signals (such as atrial fibrillation waves) even outperforms current medical clinical devices (Figure 19b–d). Soon afterward, Chan et al. proposed a van der Waals assembly idea for the preparation of OSSC transistors by sequentially transferring high‐quality organic monolayer crystals and prepatterned source/drain electrodes.^[^ ^81^ ^]^ They also achieved a small threshold voltage of −1.5 V and subthreshold swing (SS) of 63 mV decade^−1^ by low surface energy phosphonic acid SAMs on high‐k AlOx dielectric. Furthermore, with this sharp switching characteristic and by integrating organic amplifiers with ECG pads by means of a 3D printed case, they presented wearable and coin cell powered ECG sensors with a signal‐to‐noise ratio of 34 dB.

#### 4.2.4 Other Application Scenarios

**Figure 20:**
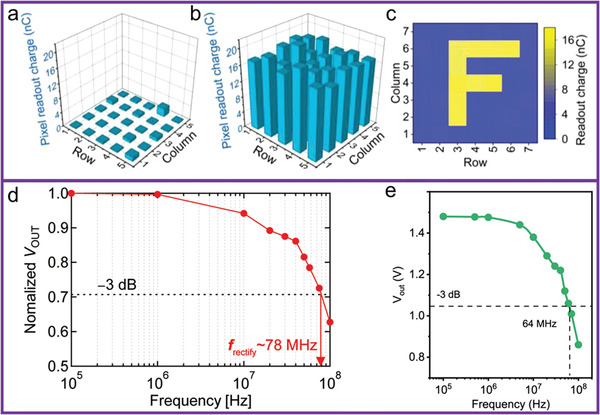
3D counter plots of the readout charges from each pixel of the memory array under a) 0 state and b) 1 state. c) Demonstration of the image “F” stored in a MFSC memory array. Yellow regions represent the 1 state and blue regions represent the 0 state, respectively. Reproduced with permission.^[^ ^82^ ^]^ Copyright 2022, Wiley‐VCH. d) Frequency dependence of the rectified output voltage (*V*_out_) normalized with respect to the value at 100 kHz. The rectifying frequency (*f*_rectify_) is extracted from the frequency at which the normalized Vout, decreases by −3 dB. Reproduced with permission.^[^ ^83^ ^]^ Copyright 2020, Wiley‐VCH. e) The output d.c. voltage as a function of frequency. Reproduced with permission.^[^ ^84^ ^]^ Copyright 2023, Springer Nature.

Semiconductor‐based nonvolatile memory is an important part of modern electronic systems, enabling long‐time information storage. Hu et al. demonstrated wafer‐scale flexible oriented molecular ferroelectric single crystal (MFSC) memory arrays. Furthermore, they investigated the uniformity of the memory function from a 5 × 5 integratable arrays. **Figure** **20**a,b shows the 3D contour maps of the “0” and “1” states of each memory cell, respectively. Thus, the high uniformity of the flexible MFSC‐based nonvolatile memory (NVM) array is proven, with a variation of about 20%. They also provided a presentation of the programmable memory performance of the NVM array (Figure 20c), confirming the capability for programmable nonvolatile memory function.^[^ ^82^ ^]^ In addition, high frequency devices are very important for long distance wireless communication. Bilayer (2L) OSSC based OFET with a channel length of 50 µm displays an outstanding mobility of more than 10 cm^2^ V^−1^ s^−1^, and low contact resistance as low as 50 Ω cm. These features of ultrathin OSSCs are essential for high‐frequency circuit applications. Furthermore, Takeya et al. showed that *V*_out_ is decreased by only 10% at the frequency for the near‐field communication of radio frequency identification (RFID) tags (13.56 MHz), which is sufficient for use as a wireless power supply for RFID tags (Figure 20d).^[^ ^83^ ^]^ Recently, MMC‐OFETs with transferred‐Pt electrodes demonstrated by Wang et al. exhibited several benchmark features, including ultralow contact resistance of 14.0 Ω cm, hole mobility of 18 cm^2^ V^−1^ s^−1^, saturation current of 28.8 µA µm^−1^ and subthreshold swing of 60 mV dec^−1^. The intrinsic cutoff frequency of their OFET is estimated to be 0.36 GHz owing to these excellent electrical properties. Their work holds promise to advance the organic semiconductors toward GHz devices (Figure 20e).^[^ ^84^ ^]^

#### 4.2.5 Integrated Circuits

**Figure 21:**
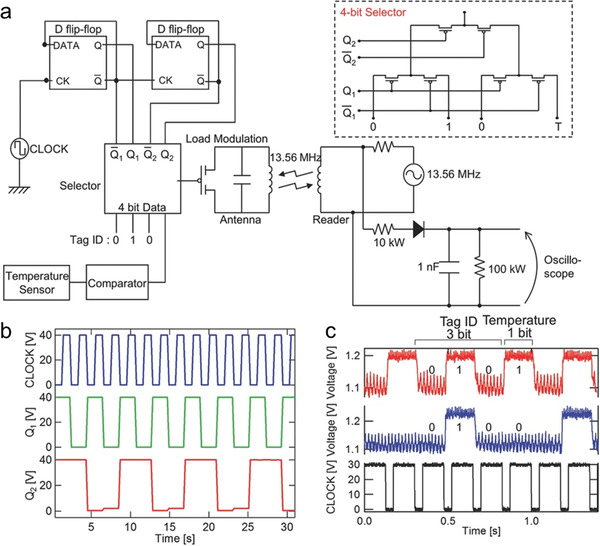
a) Left: A schematic of the RFID prototype and (right) a circuit diagram of the 4‐bit selector. b) The operation of a 2‐bit binary counter fabricated on EPRIMA AL, composed of two DFFs connected in series. Q1 and Q2 indicate the output signals from the CLOCK‐input DFF and the subsequent DFF, respectively. c) The signal received at the reader oscilloscope at two different temperatures. The top and middle signals show the output when the temperature is above and below 50 °C, respectively. The bottom signals represent the CLOCK. Reproduced with permission.^[^ ^85^ ^]^ Copyright 2017, Wiley‐VCH.

The next step in the development of electronics is presently envisioned to be the establishment of robust manufacturing technologies for integrated electronics, such as plastic sensor films and RFID tags. Yamamura et al. used the obtained single crystal film to prepare a D‐flip‐flop based on NOR logic and a binary counter obtained by connecting two D‐flip‐flops in series.^[^ ^85^ ^]^ These devices can perform logical operations normally. They combined the binary counter with a 4‐bit selector, a temperature sensor, a comparator, a load‐modulation transistor, and an antenna to demonstrate a prototypical RFID tag that can be used to detect temperature and have specific identifiable information. Among them, the combination of a binary counter and a data selector can convert a four‐bit parallel input signal into a serial output signal. The first three input “010” are generated by the input terminal being grounded or connected to a high level, as the identity information of the tag. The fourth digit of the input signal is generated by the temperature sensor: when the detected temperature is higher than 50 °C, the input signal is 1; when the detected temperature is lower than 50 °C, the input signal is 0. Finally, the tag modulates the generated serial signal and sends it wirelessly to the signal receiver. The signal receiver receives the signal and can obtain the identity and temperature information about the tag (**Figure** **21**a–c). This work advances the research of OSSC circuits to practical applications, and also shows the advantages of OSSCs as integrated circuit materials.

## 5 Summary and Outlook

**Figure 22:**
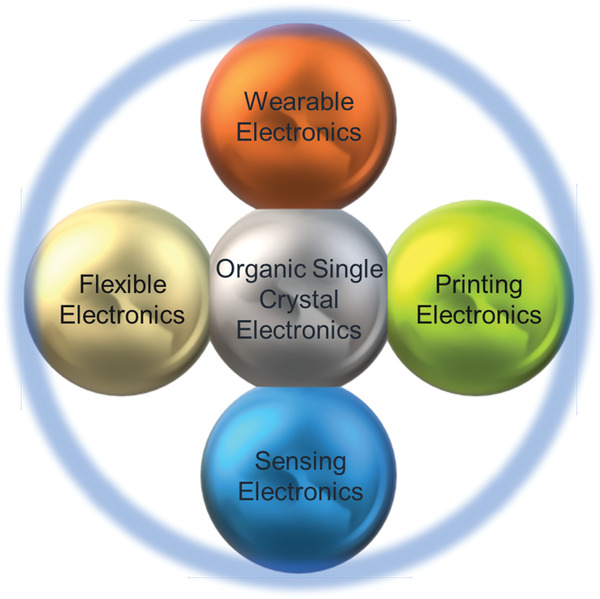
Application branches of OSSC electronics.

OSSCs patterning is essential for the practical application of organic electrical devices. In recent years, the rapid progress made in OSSC patterning and crystal growth methods has propelled the realization of a diverse range of organic electronic applications (**Figure** **22**). In this review, we focus on the recently developed techniques for controllable preparation of OSSCs arrays, and summarize their device applications. Despite all the progress achieved now, there are still many issues to be addressed before practical applications in large‐scale industrial production: 1) Most of the OSSC arrays reported so far can only be oriented in one certain direction, controlled multidirectional orientation required for complex integrated circuits remains difficult. Moreover, anisotropic optoelectronic properties of OSSCs make the more accurate and controllable patterning techniques urgently needed. 2) In addition to directional control, thickness control is equally important for large‐scale integration of OSSC arrays, because the performances of organic optoelectronic devices are closely related to the thickness of the active layer. Developing patterning techniques that can achieve both orientation and thickness control is essential. 3) The currently reported patterning techniques are more or less dependent on the molecular structures and substrate properties. A universal process compatible with any organic molecule and substrate is still lacking. For example, the organic solvents involved patterning procedures are not suitable to the flexible polymer substrates, which hinders its application in next generation wearable and stretchable devices. 4) Although MMCs have demonstrated considerable potential and benefits in the construction of organic circuits, the preparation of MMCs in arrays remains challenging and few reports have been published.

In terms of the challenges encountered above, more efforts (including but not limited to) should be devoted in following several points. 1) The evolution of fluid dynamics of solvent and molecular dynamics of solute molecules during the preparation of OSSCs are the essence of the growth process of OSSCs, so gaining deeper understanding of the fluid dynamics and molecular dynamics related to OSSCs growth will strongly promote the improvement of patterning process. 2) Developing universal method for high‐quality OSSCs with homogeneous thickness and identical orientation toward various type of substrates, and enhancing the uniformity of device performance through device engineering would propel their practical application. 3) Developing methods to realize controlled growth of OSSC at the desired specific locations with preferred orientation of OSSC is one of the critical control segments. Besides, developing and selecting OSSC materials with weak anisotropic charge transport property is an alternative solution, and it represents another outstanding shortcut to promote OSSCs to practical applications. 4) Improving the resolution of single component OSSC patterning and the integration of multicomponent OSSCs would advance high‐density integration of device arrays and construction of multifunctional devices. 5) MMCs have provided substantial benefits in high‐performance OFETs, therefore, developing low‐voltage, low‐power consumption, and miniaturized integrated circuits based on flexible MMC arrays is urgently needed.

## Conflict of Interest

The authors declare no conflict of interest.

## Author Contributions

The manuscript was written through contributions of all authors. All authors have given approval to the final version of the manuscript.
